# A Possible Social Support Paradox in Adolescent Digital Risk: Online Hate Exposure, Cyberviolence Acceptance, and Divergent Peer–Family Contexts

**DOI:** 10.3390/bs16101796

**Published:** 2026-10-01

**Authors:** Xin-Ran Li, Pil-Tae Hong

**Affiliations:** 1 School of Science and Education, Yancheng Teachers University, Yancheng 224002, China; hawa2022@163.com; 2 Graduate School of Business, Sogang University, Seoul 04107, Republic of Korea

**Keywords:** online hate exposure, cyberviolence acceptance, online risk behavior, online peer support, perceived family support, adolescents, social facilitation, moderated mediation

## Abstract

Social support is generally assumed to protect adolescents from digital risk, yet its associations may differ by source and context. Using national data from 9296 South Korean adolescents, this study examined associations among online hate exposure (OHE), cyberviolence acceptance (CVA), online risk behavior (ORB), online peer support (OPS), and perceived family support (PFS). OHE was positively associated with CVA and ORB, with a significant indirect association between OHE and ORB through CVA. OPS was positively associated with ORB, whereas PFS was negatively associated with ORB and attenuated the OPS–ORB association. The negative PFS–ORB association was weaker at higher CVA. However, explained variance was modest and both interactions were very small. The findings suggest a possible social support paradox but require longitudinal replication.

## 1. Introduction

Digital environments have become important developmental settings in which adolescents obtain information, maintain relationships, learn social norms, and experiment with behavior. Research has therefore moved beyond examining screen time alone toward the content adolescents encounter, their specific online activities, and the social contexts in which digital engagement occurs (Odgers & Jensen, 2020; Throuvala et al., 2019; Valkenburg & Peter, 2013; Valkenburg et al., 2022). Recent studies have focused on harmful content, cyberbullying, online hate, problematic social media use, and online risk behavior because their developmental consequences depend substantially on what adolescents experience and how they respond to it (Agyapong-Opoku et al., 2025; Pazdur et al., 2025; L. Wang et al., 2026). Understanding these processes is developmentally important because online risk behavior can expose adolescents to subsequent victimization, psychological distress, and other threats to digital safety and well-being.

Online hate exposure has become a particular concern. Adolescents may encounter hostility targeting nationality, ethnicity, religion, gender, appearance, disability, sexual minority status, or other personal and social identities even without deliberately seeking such material. Reviews associate these encounters with psychological distress, discriminatory attitudes, cyberaggression, and other harmful online activities (Jiménez-Díaz & Del Rey, 2025; Kansok-Dusche et al., 2023; Kowalski et al., 2014). Research also distinguishes passive exposure from intentionally searching for, sharing, or producing cyberhate, suggesting different levels of behavioral involvement (Bedrosova et al., 2023; Jiménez-Díaz & Del Rey, 2025). The observed overlap between cyberhate and cyberbullying further indicates that exposure to harmful content may be connected with broader patterns of online risk behavior (Bedrosova et al., 2022; Fulantelli et al., 2022).

Recent research has increasingly examined the cognitive processes through which such exposure becomes associated with behavior. Adolescents do not automatically reproduce every behavior they encounter; their responses depend on how online conduct is interpreted and evaluated. Repeated exposure may normalize hostile communication, reduce its perceived seriousness, and weaken resistance to cyberviolence. Moral disengagement, empathy, normative beliefs, online disinhibition, and acceptance of hate speech have consequently been examined as mechanisms linking the observation of online aggression with subsequent involvement (Bandura, 2002; Celuch et al., 2022; Suler, 2004; Wachs et al., 2022; X. Wang et al., 2022). These developments suggest that adolescents’ acceptance of cyberviolence may provide a cognitive pathway connecting online hate exposure with risk behavior.

Adolescent digital behavior is also embedded in peer and family environments. Online peers can provide belonging, emotional assistance, and opportunities for help-seeking, but peer approval and group norms may also encourage conformity and risk-taking (Marino et al., 2020; Nesi et al., 2018; Xu et al., 2023). Experimental research indicates that peer presence and approval heighten adolescents’ responsiveness to social rewards (Chein et al., 2011; Sherman et al., 2016). Family support provides a different social context by offering emotional security, communication, and guidance in managing harmful online experiences. Supportive family environments are generally associated with reduced exposure to online threats, lower cyberviolence involvement, and safer digital behavior (Lahti et al., 2024; Paek et al., 2023; Wright et al., 2021). However, peer and family support may not operate uniformly. The benefits of support can vary across sources and risk conditions (Rueger et al., 2016), and peer support can accompany greater online participation, whereas family support is more consistently protective (Babilonová et al., 2024).

Limited research has examined OHE, CVA, OPS, and PFS within one model of adolescent online risk. We use social support paradox as a provisional label for the possibility that support from different sources shows different associations with risk. It does not imply that support is inherently harmful.

The SOR framework organizes the proposed OHE–CVA–ORB sequence. Social facilitation is used only as a possible interpretation of the OPS–ORB association, and a protective-reactive perspective informs the conditional role of PFS. The study does not directly test peer co-presence or risk-specific reinforcement.

Using nationally collected data, the study evaluates direct, indirect, and conditional associations among digital exposure, cognitive acceptance, and peer and family contexts. Its contribution is the integration of these previously separated processes within one model.

## 2. Theoretical Background and Hypothesis Development

### 2.1. An Integrated SOR Framework

The SOR framework proposes that environmental stimuli influence behavior by altering individuals’ internal cognitive or affective states (Mehrabian & Russell, 1974). The framework has increasingly been applied to digital settings, where online content, interface characteristics, and social cues shape users’ evaluations before producing behavioral responses (Xia et al., 2025). It is particularly relevant to adolescents because their normative judgments and behavioral regulation remain sensitive to repeated digital and interpersonal experiences.

In the proposed model, OHE is the stimulus, CVA the cognitive state, and ORB the response. OPS and PFS represent social contexts surrounding this sequence. SOR provides the organizing framework; the other perspectives are used only to interpret specific associations.

### 2.2. Online Hate Exposure, Cyberviolence Acceptance, and Online Risk Behavior

Repeated exposure to online hate may normalize hostile communication and reduce adolescents’ sensitivity to its harmfulness. Online environments make hateful expressions visible, repeatable, and socially observable, potentially creating the perception that such conduct is common or tolerated. Studies associate exposure to cyberhate and harmful online content with moral disengagement, diminished empathy, cyberaggression, and broader online risks (Fulantelli et al., 2022; Jiménez-Díaz & Del Rey, 2025; Kansok-Dusche et al., 2023; Wachs et al., 2022). Adolescents who repeatedly encounter such content may consequently become more accepting of cyberviolence.

**H1.**  *Online hate exposure is positively associated with cyberviolence acceptance.*

OHE may also be directly associated with ORB. Harmful content can increase familiarity with risky behavioral scripts and create the impression that aggressive or unsafe conduct is widespread within digital communities. Repeated exposure may thereby lower behavioral inhibition even when its effects are not fully transmitted through conscious cognitive acceptance. Previous research connecting harmful-content exposure with cyberhate and cyberbullying perpetration supports this relationship (Bedrosova et al., 2022; Paek et al., 2023).

**H2.**  *Online hate exposure is positively associated with online risk behavior.*

CVA reflects the extent to which adolescents regard cyberviolence as permissible, understandable, or insufficiently serious. Such permissive judgments can weaken internal restraints against harmful digital conduct and increase adolescents’ willingness to engage in risky online activities. This expectation is consistent with findings linking moral disengagement, permissive norms, and online disinhibition with cyberaggression (Celuch et al., 2022; Wachs et al., 2022; X. Wang et al., 2022).

**H3.**  *Cyberviolence acceptance is positively associated with online risk behavior.*

### 2.3. Online Peer Support and Social Facilitation

Adolescent online behavior occurs within peer networks that provide belonging, assistance, feedback, and behavioral reinforcement. OPS can perform beneficial relational functions, particularly by providing encouragement and rapid assistance. However, its behavioral implications depend on the types of activities and responses that are already available in the online environment.

Social facilitation theory proposes that others’ presence can increase dominant responses (Zajonc, 1965). However, OPS measures general approval, responsiveness, and encouragement—not peer presence during risky acts or approval of those acts. Social facilitation is therefore only a tentative interpretive lens. Greater online engagement, network size, or opportunity may also explain the OPS–ORB association.

Accordingly, the analysis tests an association between general online peer responsiveness and ORB, not a causal social-facilitation mechanism.

**H4.**  *Online peer support is positively associated with online risk behavior.*

### 2.4. The Protective and Conditional Role of Perceived Family Support

PFS provides adolescents with emotional security, behavioral guidance, and assistance in managing harmful online experiences. Supportive family environments can strengthen self-regulation, facilitate communication about digital difficulties, and provide resources outside peer-centered online settings. Previous studies generally associate family support with reduced exposure to online threats, lower cyberviolence involvement, and safer online behavior (Lahti et al., 2024; Paek et al., 2023; Wright et al., 2021).

**H5.**  *Perceived family support is negatively associated with online risk behavior.*

The negative PFS–ORB association may be weaker when CVA is high. This pattern does not imply that family support creates risk.

**H5a.**  *PFS moderates the CVA–ORB association, such that the negative PFS–ORB association becomes weaker as CVA increases.*

PFS may operate differently in relation to online peer support. Adolescents with stronger family support have access to emotional and regulatory resources outside their online peer networks. They may therefore be less dependent on peer approval and better able to regulate peer-facilitated online behavior. PFS is consequently expected to attenuate the positive association between OPS and ORB.

**H5b.**  *Perceived family support moderates the positive association between online peer support and online risk behavior, such that the association becomes weaker at higher levels of perceived family support.*

### 2.5. The Mediating Role of Cyberviolence Acceptance

The SOR framework proposes that an environmental stimulus influences behavior partly through an internal psychological state. Accordingly, OHE may be associated with ORB because repeated exposure increases adolescents’ acceptance of cyberviolence, which subsequently weakens their resistance to risky online conduct. Because OHE can also be associated with behavior through behavioral imitation, emotional arousal, or other unmeasured mechanisms, CVA is expected to provide a partial rather than exclusive pathway.

**H6.**  *Cyberviolence acceptance mediates the positive association between online hate exposure and online risk behavior.*

### 2.6. The Conditional Indirect Role of Perceived Family Support

Together, H1 and H5a imply that the indirect OHE–CVA–ORB association may vary across PFS levels.

**H7.**  *Perceived family support moderates the indirect association between online hate exposure and online risk behavior through cyberviolence acceptance, such that the positive indirect association becomes stronger at higher levels of perceived family support.*

**Figure 1:**
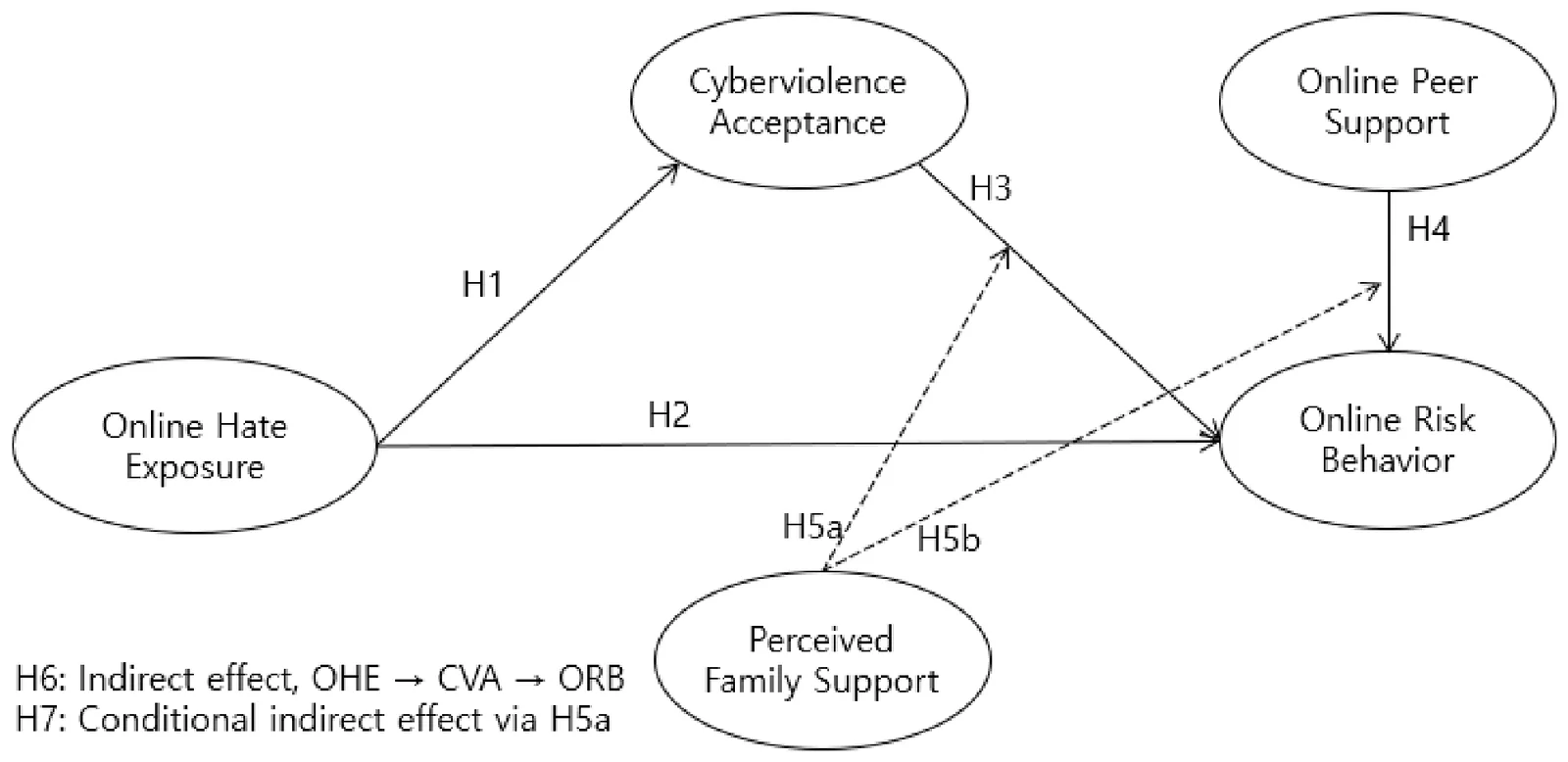
Proposed Research Model.

The model evaluates direct associations, statistical mediation, moderation, and moderated mediation (Figure 1). Because the data are cross-sectional, it does not establish temporal or causal ordering.

## 3. Materials and Methods

### 3.1. Measures

Items were drawn from the youth questionnaire of the 2025 National Survey on Cyberviolence (National Information Society Agency, 2025). The five constructs were not pre-existing scales validated for this model; the authors grouped relevant items according to the construct definitions. The original Korean items were translated into English by the authors, and the English translations were subsequently reviewed by a native English speaker to ensure linguistic accuracy and clarity.

Online Hate Exposure was measured using 12 items assessing the frequency with which adolescents encountered digital hate expressions based on nationality or race, religion, sexual minority status, disability, gender, physical appearance, multicultural background, age, income, educational background, region, and political orientation. The original response categories were 9 (no exposure), 1 (once or twice a year), 2 (once or twice every six months), 3 (once or twice a month), and 4 (almost every day). The no-exposure category was recoded from 9 to 0, producing a scale ranging from 0 to 4. Higher values indicated more frequent exposure to online hate expressions.

Cyberviolence Acceptance was assessed with five items examining whether cyberviolent behavior was regarded as acceptable when it was unintentional, caused little harm, targeted someone perceived as deserving, produced relational or institutional benefits, or occurred under unavoidable circumstances. Responses ranged from 1 (strongly disagree) to 4 (strongly agree), with higher values representing greater acceptance of cyberviolent conduct.

Online Risk Behavior was measured using four items selected from the survey to represent adolescents’ engagement in potentially harmful online activities. The items assessed accepting proposals from people first encountered online, accepting friend requests from strangers, engaging in sexual conversations with unknown individuals, and intentionally accessing illegal adult websites. Responses were recorded on a four-point scale ranging from 1 (not at all true) to 4 (very true), with higher scores indicating greater involvement in risky online behavior.

Online Peer Support was assessed with three items capturing whether online friends expressed approval of the respondent’s posts, responded promptly to messages, and provided encouragement when the respondent experienced difficulties. Perceived Family Support was measured with three items assessing whether family members encouraged the respondent’s decisions, showed interest in their school life, and listened attentively to their questions. Both constructs were measured on four-point scales ranging from 1 (not at all true) to 4 (very true), with higher scores indicating stronger perceived support.

All constructs were initially specified reflectively because the indicators were used as manifestations of the underlying constructs represented in the proposed model. However, this specification is less straightforward for OHE and ORB. The OHE indicators represent exposure across distinct hate targets, whereas the ORB indicators capture heterogeneous forms of online risk behavior. Accordingly, these two constructs may also be conceptualized as composite indices rather than strictly interchangeable reflective indicators. We therefore treat the reflective specification as an operational modeling choice and interpret the results for OHE and ORB with this measurement limitation in mind.

### 3.2. Data Source and Participants

This study used publicly available, anonymized secondary data from the youth component of the 2025 National Survey on Cyberviolence conducted by the National Information Society Agency (NIA), Republic of Korea. The survey is an official nationwide statistical investigation designed to monitor adolescents’ digital activities, exposure to cyberviolence, online experiences, and surrounding social environments. Its target population consisted of South Korean students from the fifth grade of elementary school through the third grade of high school who used the internet or smartphones.

Participants were selected through a two-stage stratified sampling procedure. Schools were first sampled within geographical strata, after which classes were selected from the participating schools. Data were collected between 1 September and 20 November 2025, through school-based, tablet-assisted survey administration. Trained field personnel explained the purpose of the survey and informed students that their responses would remain confidential.

All personally identifiable information was removed before the public-use dataset was released. The present study was based exclusively on secondary analysis of these publicly available, anonymized data. The authors were not involved in the original data collection, had no direct contact with participants, and had no access to personally identifiable information.

The final dataset comprised 9296 students, including 3124 elementary school students, 3164 middle school students, and 3008 high school students. The detailed demographic characteristics of the participants, including gender, school level, and average daily internet use, are presented in Table 1. The public-use dataset contained no missing responses for the variables included in the present analyses; therefore, no missing-data imputation or case deletion was required.

### 3.3. Analytical Strategy

The model was estimated in SmartPLS 4.1.1.4, with SPSS 26.0 used for data processing. PLS-SEM was selected because the study aimed to evaluate structural relationships together with out-of-sample predictive relevance and conditional effects using a large secondary dataset composed primarily of restricted-response survey indicators. Mediation and moderation alone were not treated as justification for PLS-SEM; CB-SEM was therefore additionally used as a supplementary robustness check of the principal direct structural relationships.

The reflective measurement models were assessed using indicator loadings, Cronbach’s alpha, rho_A, composite reliability, and average variance extracted. Discriminant validity was evaluated using the HTMT ratio and the Fornell–Larcker criterion (Fornell & Larcker, 1981; Henseler et al., 2015). Potential common method bias was examined using Harman’s single-factor test and full-collinearity VIFs (Kock, 2015).

The structural model was evaluated using inner VIF values, standardized path coefficients, coefficients of determination (R^2^), and effect sizes (f^2^). Statistical significance was assessed through two-tailed bootstrapping with 5000 resamples and 95% percentile confidence intervals. The indirect effect of OHE on ORB through CVA was estimated to test mediation. Interaction terms were then introduced to determine whether PFS moderated the relationships of CVA and OPS with ORB. Significant interactions were examined through conditional effects at low (−1 SD), mean, and high (+1 SD) levels of PFS. Gender, school level, and average daily internet use were additionally included as controls for both CVA and ORB because these characteristics may affect adolescents’ opportunities to encounter harmful content and engage in online risk behavior. The control-variable model was estimated separately to determine whether the hypothesized relationships remained stable after accounting for these characteristics. Gender was dummy-coded with boys as the reference group; school level was represented by two dummy variables with elementary school as the reference group; and average daily internet use was represented by four dummy variables with less than 1 h per day as the reference group. The index of moderated mediation was calculated for each bootstrap sample by multiplying the OHE–CVA coefficient by the PFS × CVA–ORB interaction coefficient. PLSpredict 4.1.1.4 was additionally used to assess out-of-sample predictive relevance relative to linear-model and indicator-average benchmarks (Shmueli et al., 2019).

Finally, CB-SEM was conducted in SmartPLS 4.1.1.4 using maximum likelihood estimation as a robustness check of the principal direct relationships. Mediation, moderation, and moderated mediation were evaluated only in PLS-SEM. Cross-method robustness was therefore assessed based on the direction and significance of the corresponding direct relationships rather than exact coefficient equivalence. All SEM analyses used unweighted individual-level data, without explicit adjustment of standard errors for school- or class-level clustering.

## 4. Results

### 4.1. Measurement Model

The adequacy of the measurement model was examined using indicator reliability, internal consistency, and convergent validity. As shown in Table 2, most indicator loadings exceeded the recommended level, while the lowest loading remained close to the acceptable threshold. All constructs demonstrated satisfactory internal consistency, and their AVE values supported convergent validity (Fornell & Larcker, 1981; Hair et al., 2019, 2022).

Discriminant validity was examined using both the HTMT ratios and the Fornell–Larcker criterion. As shown in Table 3 and Table 4, the results confirmed that Online Hate Exposure (OHE), Cyberviolence Acceptance (CVA), Online Risk Behavior (ORB), Online Peer Support (OPS), and Perceived Family Support (PFS) were empirically distinct from one another (Fornell & Larcker, 1981; Henseler et al., 2015).

Harman’s test and full-collinearity VIFs were used as preliminary diagnostics. Neither procedure can establish that common method bias is absent.

### 4.2. Structural Model and Direct Effects

The model explained 5.4% of CVA and 10.9% of ORB, indicating limited explanatory capacity. It addresses a focused set of associations rather than providing a comprehensive explanation or individual prediction of online risk.

The structural-path results are reported in Table 5. Online hate exposure was positively associated with cyberviolence acceptance, supporting H1. It was also positively related to online risk behavior, providing support for H2. Cyberviolence acceptance showed a positive relationship with online risk behavior, supporting H3. These findings indicate that exposure to hateful online content is associated with risky online conduct both directly and through adolescents’ cognitive acceptance of cyberviolent behavior.

Online peer support was positively associated with online risk behavior, supporting H4. This result suggests that responsive interactions within online peer contexts do not necessarily operate as an exclusively protective resource. The positive association is consistent with the possibility that such environments make already accessible online behaviors easier to enact. By contrast, perceived family support was negatively associated with online risk behavior, supporting H5 and indicating an overall negative association with ORB. Accordingly, all hypothesized direct associations were statistically significant in the predicted directions, although their effect sizes were small.

### 4.3. Mediation Analysis

Table 6 presents the indirect and total effects. Online hate exposure had a significant indirect association with online risk behavior through cyberviolence acceptance. The direct association between online hate exposure and online risk behavior also remained significant after cyberviolence acceptance was included in the model. Because the direct and indirect effects were significant and operated in the same direction, the results indicate complementary partial mediation.

The findings suggest that greater exposure to online hate is associated with stronger acceptance of cyberviolent conduct, which is in turn associated with greater online risk behavior. The mediation hypothesis, H6, was therefore supported.

### 4.4. Moderating Effects of Perceived Family Support

PFS statistically conditioned the CVA–ORB and OPS–ORB associations. Both interactions were very small (f^2^ = 0.003) and should therefore be interpreted cautiously.

Table 7 clarifies the interaction directions. The positive CVA–ORB association became stronger as PFS increased. Viewed from the reverse conditional perspective, however, PFS remained negatively associated with ORB across all three levels of CVA, although this negative association became progressively weaker as CVA increased (β = −0.214 at low CVA, β = −0.166 at mean CVA, and β = −0.119 at high CVA; all *p* < 0.001). Thus, the interaction does not indicate that family support increased risk; rather, its negative association with ORB was attenuated at higher levels of CVA.

The positive OPS–ORB association became weaker as PFS increased. Given the very small interaction effect, this pattern should be interpreted cautiously and requires replication.

**Figure A1:**
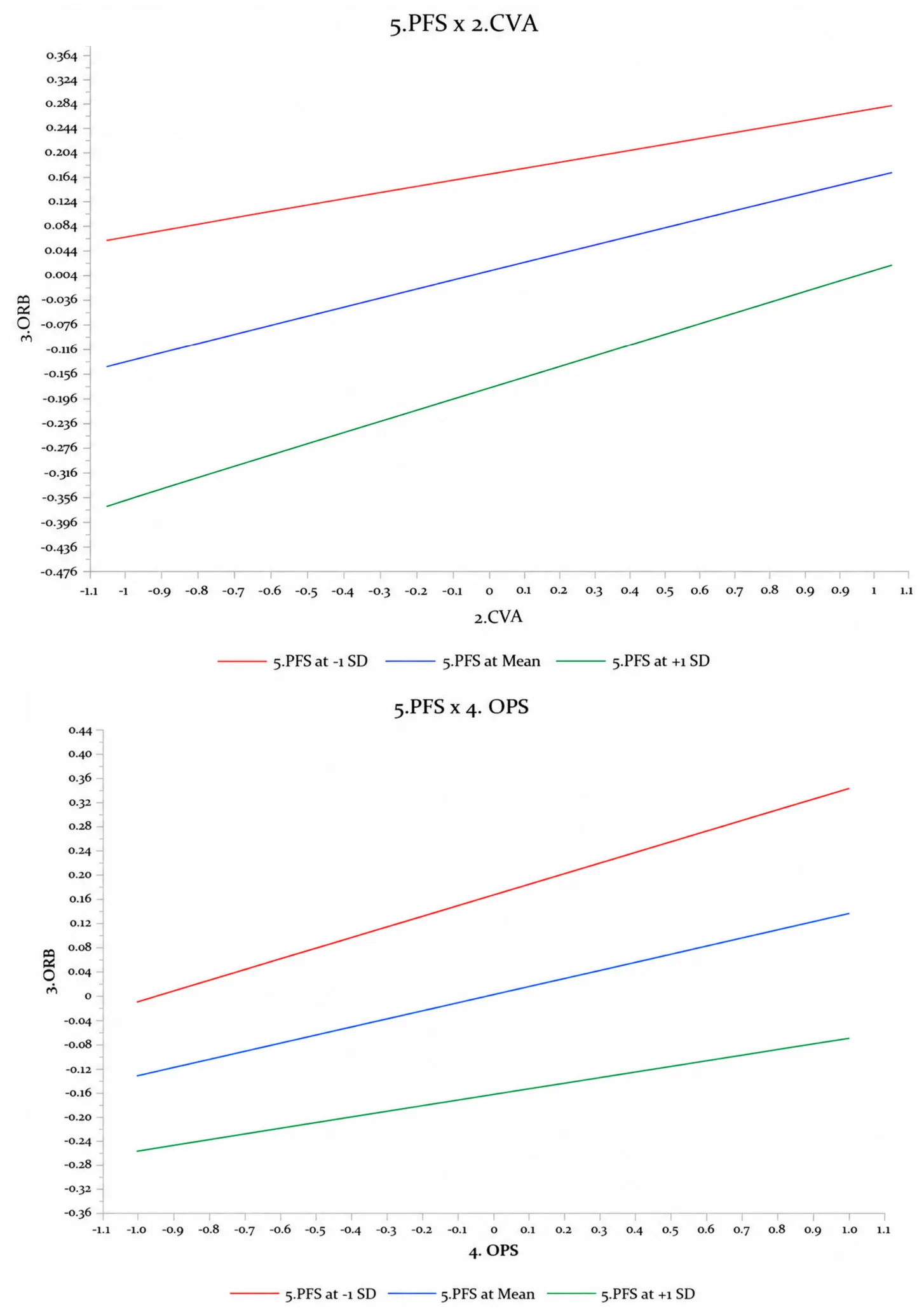
Simple-slope plots.

Appendix A Figure A1 displays these conditional patterns without implying large effects.

### 4.5. Conditional Indirect Effects and Moderated Mediation

Panel B of Table 7 reports the conditional indirect effects of online hate exposure on online risk behavior through cyberviolence acceptance. The indirect effect became progressively stronger across increasing levels of perceived family support. More importantly, the bootstrap confidence interval for the index of moderated mediation did not include zero. This result indicates that the indirect association between online hate exposure and online risk behavior through cyberviolence acceptance varied significantly according to perceived family support.

The moderated mediation index was statistically significant but small and depended on a very small interaction. It indicates a limited conditional pattern, not a practically large process.

### 4.6. Predictive Assessment

The model’s out-of-sample predictive performance was evaluated using the PLSpredict procedure. As shown in Table 8, the Q^2^_predict_ values for both endogenous constructs were positive, indicating some predictive relevance for cyberviolence acceptance and online risk behavior.

Positive **Q^2^_predict_** values indicated some predictive relevance, but the linear-model benchmark produced lower errors for most indicators. The model’s main contribution is explanatory; it is not suitable for individual risk screening.

### 4.7. Robustness Assessment

As an initial sensitivity check, the structural model was re-estimated after controlling for gender, school level, and average daily internet use. All focal direct and interaction paths reported in Table 5 retained their original directions and statistical significance after adjustment. The adjusted coefficients and 95% confidence intervals are reported in Supplementary Table S1.

CB-SEM was subsequently conducted as a supplementary robustness assessment of the principal direct structural relationships. The model demonstrated an acceptable fit (RMSEA = 0.068, SRMR = 0.042, CFI = 0.915, and TLI = 0.904). The CB-SEM analysis reproduced the positive relationships of OHE with CVA and ORB, the positive relationships of CVA and OPS with ORB, and the negative relationship between PFS and ORB. All corresponding direct relationships retained their statistical significance. Because CB-SEM was used only as a supplementary robustness check of the direct structural relationships, mediation, moderation, and moderated mediation were evaluated using PLS-SEM.

### 4.8. Summary of Hypothesis Testing

The estimated directions were statistically consistent with the hypotheses. However, the direct associations were small, both interactions were very small, and explained variance was modest.

## 5. Discussion

### 5.1. Online Hate Exposure, Cognitive Acceptance, and Risk Behavior

This study examined how online hate exposure is associated with adolescents’ online risk behavior through cognitive evaluation and social context. The results were consistent with the proposed SOR sequence. OHE was positively associated with both CVA and ORB, while CVA was positively associated with ORB, with a significant indirect association between OHE and ORB through CVA.

These findings extend research connecting harmful-content exposure with cyberhate, cyberbullying, and other digital risks (Bedrosova et al., 2022; Fulantelli et al., 2022; Kansok-Dusche et al., 2023; Paek et al., 2023). Recent evidence from Korean adolescents similarly shows that exposure to risky online content is associated with moral disengagement and cyberaggression, while media literacy can weaken parts of this cognitive process (Lee & Jun, 2024). Longitudinal research also identifies moral disengagement as a mechanism through which harmful online communication may develop into cyberaggression (Falla et al., 2021).

Exposure therefore does not necessarily determine behavior automatically. Its behavioral relevance depends partly on whether adolescents come to regard cyberviolence as normal, permissible, or insufficiently serious. The remaining direct association between OHE and ORB suggests that other mechanisms, including behavioral imitation, emotional arousal, online disinhibition, or self-selection into risky digital environments, may also be involved (Wachs et al., 2022; X. Wang et al., 2022).

The results support the application of SOR to adolescent digital behavior: Within the proposed SOR framework, OHE is conceptualized as a harmful environmental stimulus, CVA as an internal cognitive state, and ORB as the behavioral response. Although the explained variance was modest, the model identifies a coherent cognitive pathway within the broader set of personal, social, and technological factors associated with online risk behavior (Pazdur et al., 2025; L. Wang et al., 2026).

### 5.2. The Social Support Paradox: Contrasting Peer and Family Contexts

The findings suggest a possible, not established, social support paradox (Carapeto et al., 2025). OPS was positively associated with ORB, whereas PFS was negatively associated with ORB. OPS did not measure risk-specific peer approval; the positive coefficient may instead reflect greater online engagement, network size, opportunity, self-selection, or broader peer-influence processes (Laursen, 2021).

PFS, by contrast, was associated with lower ORB. Supportive family relationships may provide adolescents with emotional security, guidance, monitoring, and regulatory resources outside their online peer networks. This result is consistent with studies associating family support, constructive parental involvement, and lower family conflict with reduced exposure to online threats and lower cyberviolence involvement (Lahti et al., 2024; Paek et al., 2023; Shao et al., 2024; Wright et al., 2021). Peer and family support should therefore not be treated as interchangeable forms of social support in adolescent digital-risk research.

### 5.3. Family Support as a Conditional Protective Resource

This conditional interpretation is consistent with resilience perspectives emphasizing that protective resources may operate differently across risk conditions (Fergus & Zimmerman, 2005). PFS weakened the positive OPS–ORB association, suggesting that supportive family environments can reduce adolescents’ susceptibility to influences arising from online peer contexts. Adolescents with stronger family support may be less dependent on online approval and better able to regulate peer-facilitated behavior. This interpretation is consistent with research identifying family communication and support as resources that reduce involvement in cyberbullying and other digital risks (Rodriguez-Rivas et al., 2022; Shao et al., 2024; Wright et al., 2021).

The negative PFS–ORB association was weaker at higher CVA. The interaction was very small and does not show that family support loses protection over time.

The conditional indirect association varied across PFS levels, but its small magnitude limits practical interpretation.

### 5.4. Predictive and Robustness Evidence

The PLSpredict results demonstrated positive predictive relevance for both endogenous constructs, although the PLS-SEM model showed limited improvement over the linear-model benchmark. The model should therefore be understood primarily as an explanation of cognitive and contextual relationships rather than as a tool for highly accurate individual prediction. This interpretation is consistent with recent reviews emphasizing the heterogeneous and multicausal nature of adolescent online risk behavior (Pazdur et al., 2025; L. Wang et al., 2026).

The CB-SEM analysis provided supplementary robustness evidence. The principal direct relationships retained their directions and statistical significance across the two estimation approaches, indicating that these direct associations were not dependent solely on PLS-SEM estimation.

## 6. Conclusions

The data were consistent with, but did not confirm, the proposed OHE–CVA–ORB sequence. OPS and PFS showed different associations with ORB, but explained variance was modest and the interactions were very small. The possible social support paradox therefore requires longitudinal replication with purpose-developed measures.

### 6.1. Theoretical Contributions

The first contribution is to distinguish peer and family support while evaluating CVA as a statistical mediator of the OHE–ORB association and PFS as a conditional correlate. This contribution concerns patterns of association, not causal processes.

Second, the study extends the SOR framework to adolescent digital risk behavior by examining cyberviolence acceptance as a cognitive pathway statistically linking online hate exposure with online risk behavior. This moves beyond treating harmful-content exposure as an exclusively direct risk factor and suggests that adolescents’ interpretations of harmful conduct may help transmit the association between digital exposure and behavior. The findings complement evidence that permissive beliefs, moral disengagement, and related cognitive processes weaken internal restraints against online aggression (Bandura, 2002; Lee & Jun, 2024; Wachs et al., 2022).

Third, the study integrates social facilitation and protective-reactive perspectives to explain why peer and family contexts operate differently. Online peer support captures a responsive peer environment rather than peers’ explicit approval of particular risky acts. Its positive association with risk behavior is nevertheless consistent with the possibility that peer responsiveness facilitates behaviors already available in adolescents’ digital settings. Perceived family support, by contrast, showed a negative association with ORB and moderated the peer-related association, although these patterns should not be interpreted causally. Social protection and cognitive risk therefore operate jointly rather than independently, providing a more conditional account than the assumption that support uniformly neutralizes digital risk.

### 6.2. Practical Implications

The study did not test interventions. Its findings only suggest that future programs could jointly examine harmful-content exposure and adolescents’ evaluations of online conduct. Future peer-based programs could test whether risk-specific norms and online reinforcement are more relevant than general peer support.

The results do not show that changing family support or CVA will reduce ORB. Future intervention studies could test supportive communication together with explicit discussion of online norms, digital literacy, and adaptive emotion regulation (Liang et al., 2026).

### 6.3. Limitations and Future Research

First, the cross-sectional design precludes causal or temporal conclusions, and reverse relationships are possible. Adolescents already engaged in online risk behavior may encounter more harmful content or develop different online peer relationships. Longitudinal research is needed to clarify these reciprocal associations.

Second, the study relied on self-reported secondary data, and the constructs were assembled from available survey items rather than instruments designed specifically for the present model. OPS also measured general peer support rather than risk-specific peer norms. The ORB construct combined four behaviors that differ in nature and severity and may have different predictors, potentially obscuring behavior-specific patterns. Moreover, because the OHE and ORB indicators were predetermined by the original survey and were not specifically designed to distinguish between reflective and formative measurement specifications, their reflective specification was retained as an analytical choice within the present measurement framework. Given the heterogeneous content of these indicators, however, this specification should not be regarded as a definitive representation of their measurement structure. Future research should test theoretically justified formative or composite specifications and assess whether the structural relationships remain robust across alternative measurement models. Accordingly, recall bias, shared-method variance, and measurement limitations remain possible, and the common-method diagnostics used here cannot rule out such bias.

Finally, the modest explained variance and very small interaction effects limit the substantive and predictive scope of the findings. In addition, because school- and class-level identifiers were not available in the secondary dataset, the clustering structure of respondents could not be reconstructed, precluding design-adjusted, cluster-robust, or multilevel sensitivity analyses. Potential clustering of students within schools or classes may therefore affect standard errors and statistical inference. The results should also not be interpreted as weighted population estimates. Future research should replicate the findings using longitudinal, purpose-designed, and design-adjusted data with school- and class-level identifiers that permit cluster-adjusted or multilevel analyses. Future research should also examine additional factors such as moral disengagement, impulsivity, parental mediation, and personality characteristics that may account for the unexplained variance.

## Figures and Tables

**Table 1:** Sample Characteristics of the Participants (N = 9296).

Demographics	Subjects	Frequency	Percentage
Gender	Boy	4902	52.7%
	Girl	4394	47.3%
School Level	Elementary School (5th & 6th Grades)	3124	33.6%
	Middle School	3164	34.0%
	High School	3008	32.4%
Average Daily Internet Use	Less than 1 h	658	7.1%
	1 h or more to less than 2 h	1712	18.4%
	2 h or more to less than 3 h	2411	25.9%
	3 h or more to less than 5 h	2390	25.7%
	5 h or more	2125	22.9%

**Table 2:** Measurement Model Reliability and Convergent Validity.

Construct	Abbreviation	Factor Loading	Cronbach’s Alpha	rho_A	rho_C	AVE
Online Hate Exposure	OHE	0.736~0.804	0.929	0.930	0.939	0.608
Cyberviolence Acceptance	CVA	0.799~0.850	0.885	0.892	0.915	0.683
Online Risk Behavior	ORB	0.685~0.753	0.707	0.705	0.819	0.531
Online Peer Support	OPS	0.719~0.904	0.811	0.889	0.877	0.705
Perceived Family Support	PFS	0.822~0.866	0.809	0.823	0.886	0.722

**Table 3:** HTMT Matrix.

Variable	1	2	3	4	5
1. OHE					
2. CVA	0.249				
3. ORB	0.246	0.272			
4. OPS	0.028	0.035	0.121		
5. PFS	0.079	0.192	0.207	0.375	

**Table 4:** Fornell–Larcker Criterion.

Variable	1	2	3	4	5
1. OHE	0.780				
2. CVA	0.232	0.827			
3. ORB	0.204	0.223	0.729		
4. OPS	0.022	0.009	0.093	0.840	
5. PFS	−0.070	−0.163	−0.157	0.278	0.850

**Table 5:** Analysis of Path Coefficients.

H	Path	β	t	*p*	Inner VIF	f^2^	95% CI	Results
H1	OHE → CVA	0.232	16.277	<0.001	1.000	0.057	0.205~0.261	Supported
H2	OHE → ORB	0.154	10.864	<0.001	1.061	0.025	0.127~0.183	Supported
H3	CVA → ORB	0.162	13.602	<0.001	1.115	0.027	0.139~0.186	Supported
H4	OPS → ORB	0.136	12.261	<0.001	1.091	0.019	0.113~0.157	Supported
H5	PFS → ORB	−0.166	14.000	<0.001	1.202	0.026	−0.190~−0.143	Supported
H5a	PFS×CVA → ORB	0.048	3.934	<0.001	1.044	0.003	0.024~0.071	Supported
H5b	PFS×OPS → ORB	−0.042	4.253	<0.001	1.082	0.003	−0.060~−0.022	Supported

NOTE. R^2^ = 0.054 for CVA and 0.109 for ORB. Effect magnitude is reported using f^2^; statistical significance should be interpreted separately from effect size.

**Table 6:** Indirect and Total Effects.

Effect Type	Path	β	STDEV	t	*p*	95% CI	Results
Specific indirect effect	OHE → CVA → ORB	0.038	0.004	10.536	<0.001	0.031~0.045	Significant
Total effect	OHE → ORB	0.192	0.015	13.142	<0.001	0.164~0.221	Significant

**Table 7:** Conditional Direct and Indirect Effects at Different Levels of Perceived Family Support. **Panel A**. Conditional direct effects. **Panel B.** Conditional indirect effects and index of moderated mediation. **Panel C.** Conditional Effects and Moderated Mediation Results.

**Panel A**						
**Path**	**PFS Level**	**β**	**STDEV**	**t**	***p***	**95% CI**
CVA → ORB	Low (−1 SD)	0.115	0.015	7.714	<0.001	0.086~0.145
CVA → ORB	Mean	0.162	0.012	13.602	<0.001	0.139~0.186
CVA → ORB	High (+1 SD)	0.210	0.019	11.143	<0.001	0.173~0.247
OPS → ORB	Low (−1 SD)	0.177	0.015	11.428	<0.001	0.146~0.207
OPS → ORB	Mean	0.136	0.011	12.261	<0.001	0.113~0.157
OPS → ORB	High (+1 SD)	0.094	0.014	6.721	<0.001	0.067~0.121
**Panel B**						
**Path**	**PFS Level**	**β/Index**	**STDEV**	**t**	***p***	**95% CI**
OHE → CVA → ORB	Low (−1 SD)	0.027	0.004	7.045	<0.001	0.020~0.035
OHE → CVA → ORB	Mean	0.038	0.004	10.536	<0.001	0.031~0.045
OHE → CVA → ORB	High (+1 SD)	0.049	0.005	9.179	<0.001	0.039~0.060
Index of moderated mediation		0.011	0.003	3.793	<0.001	0.005~0.017
**Panel C**						
**Path**	**CVA Level**	**β**	**STDEV**	**t**	***p***	**95% CI**
PFS → ORB	Low (−1 SD)	−0.214	0.015	13.870	<0.001	−0.244~−0.184
PFS → ORB	Mean	−0.166	0.012	13.999	<0.001	−0.190~−0.143
PFS → ORB	High (+1 SD)	−0.119	0.018	6.474	<0.001	−0.155~−0.083

**Table 8:** PLSpredict Assessment.

Construct	Q^2^_predict_	RMSE	MAE	Comparison with LM	Predictive Assessment
CVA	0.053	0.973	0.818	LM generally lower	Limited
ORB	0.081	0.959	0.725	LM generally lower	Limited

## Data Availability

The data used in this study were obtained from the 2025 National Survey on Cyberviolence administered by the National Information Society Agency of the Republic of Korea. The anonymized public-use dataset is available through the official public data portal in accordance with the agency’s data-access and use conditions: https://www.data.go.kr/data/15106136/fileData.do (accessed on 29 August 2026).

## Supplementary Materials

The following supporting information can be downloaded at: https://www.mdpi.com/article/10.3390/bs16101796/s1, Table S1: Robustness of Focal Structural Relationships After Adjustment for Gender, School Level, and Average Daily Internet Use.

## Abbreviations

The following abbreviations are used in this manuscript:

OHE	Online Hate Exposure
CVA	Cyberviolence Acceptance
ORB	Online Risk Behavior
OPS	Online Peer Support
PFS	Perceived Family Support

## Appendix A

**Table A1:** List of Constructs and Items.

Construct	Item	Description	Reference
Online Hate Exposure	OHE1	Frequency of encountering digital hate expressions targeting nationality or race	National Information Society Agency (2025)
	OHE2	Frequency of encountering digital hate expressions targeting religion	
	OHE3	Frequency of encountering digital hate expressions targeting sexual minorities	
	OHE4	Frequency of encountering digital hate expressions targeting persons with disabilities	
	OHE5	Frequency of encountering digital hate expressions targeting gender	
	OHE6	Frequency of encountering digital hate expressions targeting physical appearance or body	
	OHE7	Frequency of encountering digital hate expressions targeting people from multicultural backgrounds	
	OHE8	Frequency of encountering digital hate expressions targeting a particular age group	
	OHE9	Frequency of encountering digital hate expressions targeting income level	
	OHE10	Frequency of encountering digital hate expressions targeting educational background or academic credentials	
	OHE11	Frequency of encountering digital hate expressions targeting a particular region	
	OHE12	Frequency of encountering digital hate expressions targeting political orientation	
Cyberviolence Acceptance	CVA1	Cyberviolence is acceptable when it occurs unintentionally or arises naturally.	National Information Society Agency (2025)
	CVA2	Cyberviolence is acceptable when it does not cause serious harm.	
	CVA3	Cyberviolence is acceptable when the target is someone who deserves it.	
	CVA4	Cyberviolence is acceptable when it benefits one’s relationships with friends or family or one’s school or work life.	
	CVA5	Cyberviolence is acceptable when it occurs under unavoidable circumstances.	
Online Risk Behavior	ORB1	I accept proposals or requests made by people whom I first met online.	National Information Society Agency (2025)
	ORB2	I accept friend requests from people I do not know online.	
	ORB3	I engage in sexual conversations with strangers online.	
	ORB4	I intentionally access illegal adult websites.	
Online Peer Support	OPS1	My online friends express approval of my posts by using functions such as ‘Like’ or other reactions.	National Information Society Agency (2025)
	OPS2	My online friends show interest in and promptly respond to my messages.	
	OPS3	When I am having a difficult time, my online friends actively send me encouraging messages through text messages or social networking services.	
Perceived Family Support	PFS1	My family encourages and supports my decisions and choices.	National Information Society Agency (2025)
	PFS2	My family asks with interest about how I am doing at school.	
	PFS3	My family listens attentively and responds even when I ask about minor matters.	
