# Supplementary material for: A Possible Social Support Paradox in Adolescent Digital Risk: Online Hate Exposure, Cyberviolence Acceptance, and Divergent Peer–Family Contexts
Source: Behav Sci (Basel). 2026 Oct 1;16(10):1796. doi: 10.3390/bs16101796 (PMC13701377; doi:10.3390/bs16101796)
Supplement: Supplementary file 1 [file behavsci-16-01796-s001.zip › behavsci-4562461-supplementary.pdf]

Supplementary Materials

Supplementary Table S1. Robustness of Focal Structural Relationships After Adjustment for Gender, School Level, and Average Daily Internet Use

Path	Adjusted β	SE	t	p	95% CI
OHE → CVA	0.216	0.014	15.286	<.001	0.188 ~ 0.244
OHE → ORB	0.139	0.014	10.096	<.001	0.112 ~ 0.166
CVA → ORB	0.135	0.012	11.270	<.001	0.111 ~ 0.159
OPS → ORB	0.122	0.012	10.295	<.001	0.098 ~ 0.145
PFS → ORB	−0.158	0.012	13.541	<.001	-0.181 ~ -0.135
PFS × CVA → ORB	0.042	0.012	3.467	<.001	0.018 ~ 0.065
PFS × OPS → ORB	−0.043	0.010	4.450	<.001	-0.062 ~ -0.024

Note. Adjusted coefficients are from the PLS-SEM model controlling for gender, school level, and average daily internet use. OHE = online hate exposure; CVA = cyberviolence acceptance; ORB = online risk behavior; OPS = online peer support; PFS = perceived family support; CI = confidence interval.
